# Osteopathic Manipulative Treatment of Herpes Zoster Ophthalmicus/Postherpetic Neuralgia

**DOI:** 10.7759/cureus.14906

**Published:** 2021-05-08

**Authors:** Mikhail Volokitin, Nazanin Izadi, Ramona Myers, Ndeye Kane Diaw, Susan Milani

**Affiliations:** 1 Osteopathic Manipulative Medicine, Touro College of Osteopathic Medicine, New York, USA

**Keywords:** postherpetic neuralgia, herpes zoster ophthalmicus, osteopathic manipulative medicine, herpes zoster virus

## Abstract

Herpes zoster (HZ) and herpes zoster ophthalmicus (HZO) are the result of reactivation of varicella-zoster virus (VZV) from a dormant condition. Although HZ symptoms typically subside after a few weeks, HZO and postherpetic neuralgia (PHN) can persist at least 90 days after the appearance of the HZ rash. Presently, there is no gold standard for a disease-modifying therapy for postherpetic neuralgia and the current treatment is focused on early intervention and management of symptoms and dermatological complications. In the present case, a 74-year-old Caucasian male initially developed severe right-sided eye pain and headache. He was diagnosed with HZO and treated with acyclovir, but later developed swelling over the right eye and skin rash over the right side of the forehead and face. He presented to the office after the acute manifestation of the infection disappeared, but the headache and scalp hypersensitivity persisted and increased. Osteopathic manipulative treatment (OMT) included correction of cranial strains, inhibition, myofascial release, balanced ligamentous tension, and facilitated positional release. In one week, the patient reported a reduction in pain from 10/10 to 2/10. Two weeks later, he reported complete resolution of his initial symptoms. There are a limited number of cases that illustrate the benefit of OMT in diminishing pain and associated symptoms in different types of neuralgias. OMT ensures the restoration of normal anatomical structure and associated function through correcting somatic dysfunction, normalization of blood supply, muscle tone, and lymphatic drainage, therefore, providing pain relief. Better documentation of case reports and more research in this area would greatly benefit the medical community. The present case demonstrates the successful treatment of PHN with OMT. OMT can be successfully used as an adjunct therapy in cases of HZ and PHN.

## Introduction

Herpes zoster (HZ) is a result of the reactivation of dormant varicella-zoster virus (VZV). HZ causes a rash in a unilateral dermatomal distribution and symptoms typically subside in a few weeks. Herpes simplex virus type [1] is defined as a neurotropic virus, as it is the source of recurrent mucocutaneous disease that stems from its ability to establish latency in peripheral sensory ganglia (PSG) and reactivate [1]. This viral 'tropism' may be a consequence of anatomical barriers, a unique transport mechanism, or three-dimensional tissue-virus-specific interactions. However, if the infection persists after its acute phase, it can lead to chronic complications such as postherpetic neuralgia (PHN) [2]. PHN is defined as pain or allodynia that persists at least three months after resolution of the rash and it is believed to be caused by nerve damage. Similarly, if the reactivation happens in the first division of the trigeminal nerve, it can lead to a condition called herpes zoster ophthalmicus (HZO) that can also have debilitating complications including keratitis and vision loss [3].

The risk of VZV reactivation as well as developing the associated complications increase in the elderly and immunocompromised individuals [2,4]. Nearly half of patients with HZ or PHN report their pain as "horrible" or "excruciating", varying in duration from a few minutes to a constant daily basis. The pain can interfere with their everyday functioning and negatively affect their quality of life [5]. Currently, there is no gold standard for a disease-modifying therapy for PHN. The current treatment recommendations are focused on early intervention, management of symptoms, and dermatological complications [4].

Osteopathic manipulative therapy (OMT) provides means for the restoration of normal anatomical structure and therefore function. There have been several cases that illustrate the benefits of OMT in diminishing a patient's pain and associated symptoms in different types of neuralgias. In the current report, we describe the case of a patient who presented with severe right-sided eye pain and headache. He was diagnosed as having PHN following HZO and was treated using OMT.

## Case presentation

A 74-year-old Caucasian male developed severe right-sided eye pain and headache after experiencing a stressful event two days prior. The patient consulted with his physician and was diagnosed with HZO on April 1, 2019. The patient was prescribed an antiviral, oral acyclovir 800mg five times a day for 10 days. However, the right-sided eye pain and headache did not subside and over the next seven days, swelling of the right eye developed. Additionally, a skin rash developed over the right side of the forehead and face. The patient was treated with a topical steroid ointment for the skin rash, blepharitis (inflammation of the upper eyelid), and conjunctivitis with steroid eye drops. 

The patient's past medical history is significant for well-controlled benign prostatic hyperplasia (BPH) and psoriasis with the last flare-up in 2015. He denied any past surgical history or recent trauma and has an unremarkable family history. The vaccination history is unknown and there is no evidence of him receiving the HZ vaccine. 

**Figure 1:**
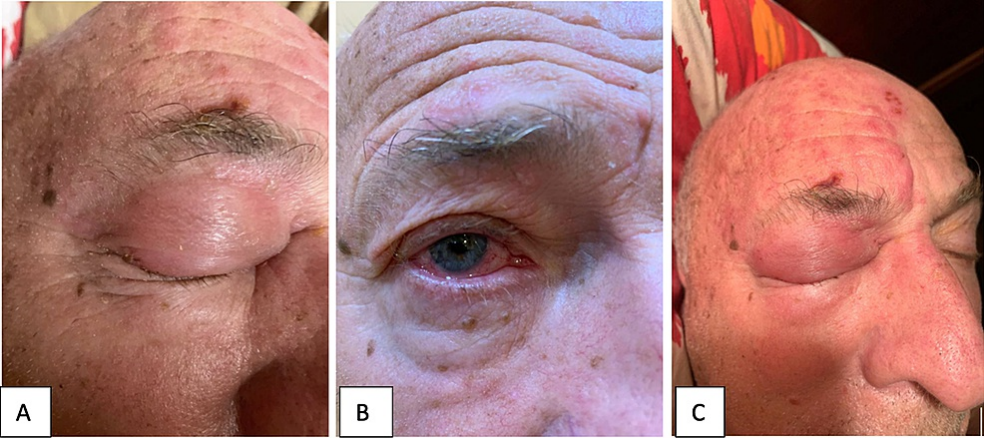
HZO presentation on the initial visit (pre-treatment). (A) Edema and inflammation of the right upper eyelid (blepharitis); (B) conjunctivitis of the right eye; (C) skin rash involving the ophthalmic division of the trigeminal nerve on the right – presenting in a unilateral dermatomal distribution. HZO: herpes zoster ophthalmicus.

The patient presented to our office six weeks after his initial symptoms, on May 16, 2019, complaining of headache and skin hypersensitivity to touch that persisted after the acute manifestations of infection disappeared. It deprived him of sleep at least for two weeks since putting his head on the pillow was excruciatingly painful. He also complained of a tingling sensation in his right arm. The patient denied any visual disturbances and did not have an ophthalmologic consultation (Figure 1).

An osteopathic structural exam (OSE) was conducted on the initial visit to reveal an extreme skin sensitivity to touch in the right greater occipital nerve distribution, increased tone in the right anterior cervical muscles: sternocleidomastoid, scalenes, and platysma; posterior cervical muscles: erector spinae, splenius capitis, semispinalis capitis, upper trapezius right>left; the right rhomboids, and pectoralis major and minor muscles. The first rib on the right was found to be in an exhaled position. The motion of the cervical spine and sternum in sagittal and coronal planes were restricted secondary to muscle hypertonicity and cervical somatic dysfunction. The patient's cranial examination presented with right occipital condyle compression, externally rotated right temporal bone, and the right side-bending/rotation strain of sphenobasilar synchondrosis (SBS).

On the initial visit, the physician used myofascial release and reciprocal inhibition to treat the hypertonic muscles and the cranial dysfunctions were treated via exaggeration. Balanced ligamentous tension (BLT) was used to restore the motion of the sternum and the first rib was treated using the facilitated positional release (FPR) technique. The patient was also instructed to do stretching and isometric exercises for the cervical and upper thoracic muscles. For more information on the techniques used, please refer to the section below.

**Figure 2:**
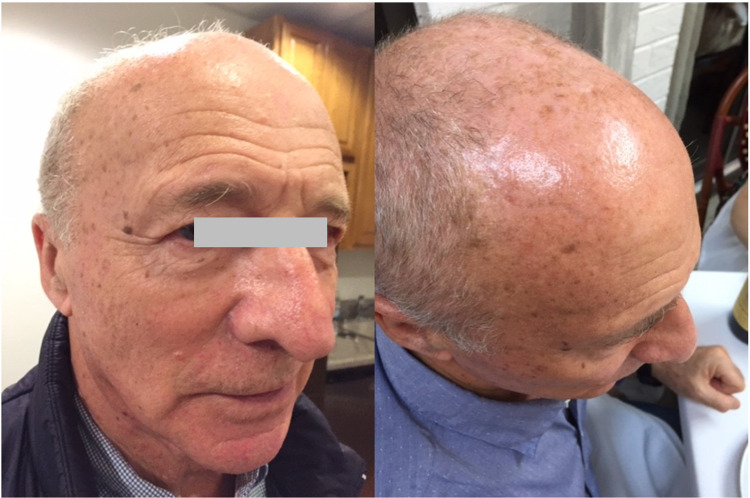
Complete resolution of patient’s symptoms 33 days after receiving osteopathic manipulative treatment.

The patient failed to appear for the first follow-up visit scheduled for June 3rd, 2019. However, he reported over the phone that his pain intensity decreased from 10/10 on his initial visit to 2/10. On the second follow-up visit two weeks later, he reported a complete resolution of the initial symptoms. On OSE, there were significant improvements in the range of motion for the cervical spine as well as the muscle tone in the affected areas. The previous somatic dysfunctions were also resolved (Figure 2).

Overview of OMTs used

Myofascial release is a form of manual medicine that combines several types of OMT to stretch and release muscle (myo) and fascia (fascial) restrictions. It helps to restore the functional balance of all tissues in the musculoskeletal system [6].

Reciprocal Inhibition is a technique that uses the reflex mechanism of reciprocal inhibition. In other words, when the antagonist muscles are contracted, the signals are conveyed to the spinal cord, and then through the reciprocal inhibition reflex arc, the agonist muscle is forced to relax [6].

The exaggeration technique used in treating the cranial somatic dysfunctions uses the inherent forces in the body to eventually increase the movement toward the ease and back to the original balance position. It is done by moving the dysfunction toward ease and adding an activating force when the ease barrier is met [6]. 

The BLT technique uses the inherent forces in the body, such as a circulatory, lymphatic, or primary respiratory mechanism to balance the tension among ligaments supporting a joint. The physician introduces a force to position the patient so that a fulcrum may be set. This fulcrum, paired with the subsequent lever action of the tissues (ligaments), combines with fluid dynamics and other factors to produce a change in the dysfunctional state [6].

FPR is a patient-passive technique with the focus on reducing abnormal muscle hypertonicity and restoring lost motion to a restricted articulation. The physician would place the body component into a neutral position, minimizing tissue and joint tension in all planes, and then add an activating force (compression or torsion) [6].

## Discussion

PHN can be potentially debilitating and impair the patient's ability to perform activities of daily living (ADL) [4, 7]. Patients are often dissatisfied with the treatment options offered for PHN [8]. Combination therapy is the common practice. Topical treatments are usually used in conjunction with oral medications. Antiviral medications (acyclovir, valacyclovir, and famciclovir) have been approved by the Food and Drug Administration for the treatment of HZ [5]. They are much more effective if they are started within the first 72 hours of the onset of the herpetic rash [5]. Usually, the patient is treated in the acute phase of HZ before the onset of PHN. If antiviral medications (or tricyclic antidepressants, which are commonly used to treat PHN) are administered during the acute phase, they can help reduce the severity and the duration of PHN [4]. Other medications that are used to treat PHN include gabapentin, lidocaine patch (5%), pregabalin, and opioids for refractory cases [4].

In some cases [9,10], the administration of an antiviral medication does not prevent the progression to PHN. For example, Pierce et al. [9] reported a case of a 95-year-old male who presented with a vesicular-bullous rash on the right side of his chest and back (T3 to T6) with associated severe pain. The patient was treated with famciclovir and prednisone for 10 days. The treatment helped to clear the rash, but PHN occurred over several dermatomes, including those originally affected. 

Surgical approach offers superficial epidermal surgical removal of a recurrent herpes simplex lesion, which prevents reinoculation of that epidermal site by the virus-laden sensory neuron [11].

OMT as performed in the present case can greatly reduce pain and limitation in range of motion in patients with PHN. Providing pain relief to a patient through OMT decreases the need for medications and the associated side effects. It may also allow the patient to avoid surgery. Several cases have been published that illustrate the benefit of OMT in diminishing a patient's pain and associated symptoms in different types of neuralgias. Hallaq and Harris [12] reported a case of postherpetic brachial neuralgia complicated by right upper monoparesis where early manipulative therapy helped to restore the function of the upper extremity. In another case described by Coffey [13], OMT was able to alleviate a severe pain in the area of the maxillary division of the trigeminal nerve in a patient with a past medical history of trigeminal neuralgia. The patient was able to decrease the daily dose of carbamazepine that was initially prescribed to manage the pain. In a study published by Richardson et al. [14], a case of Notalgia Paresthetica was discussed, and the patient stated an immediate as well as continued improvement in the symptoms after receiving manipulative treatment. OMT also showed to be superior in relieving the pain compared to NSAIDs or physical therapy in a patient with L4 radiculopathy reported by Lewis and Summers [15]. Origo and Tarantino [16] also described a case in which OMT was effective in improving disability and activities of daily living in a patient with pudendal neuralgia. These published cases are just a few examples that demonstrate the benefits of OMT in treating different cases of neuralgia. More research is needed to investigate the extent of OMT efficacy and provide additional information regarding the mechanisms involved.

## Conclusions

The presented case has demonstrated the successful treatment of PHN with OMT. There are no available PHN cases in the literature and in general, there is a lack of case studies where OMT is used in the management of neuralgias. OMT ensures the restoration of normal anatomical structure and therefore function through correcting somatic dysfunction, providing optimal conditions for body to heal itself, normalization of blood supply, peripheral nervous system regulation, muscle tone and lymphatic drainage therefore providing pain relief and reducing the need for additional medications. OMT can be successfully used as an adjunct therapy in cases of herpes zoster and postherpetic neuralgia.

## References

[1] Oxman MN (2009). Herpes zoster pathogenesis and cell-mediated immunity and immunosenescence. J Am Osteopath Assoc.

[2] Weaver BA (2009). Herpes zoster overview: natural history and incidence. J Am Osteopath Assoc.

[3] Sampathkumar P, Drage LA, Martin DP (2009). Herpes zoster (shingles) and postherpetic neuralgia. Mayo Clin Proc.

[4] Delengocky T, Bui CM (2008). Complete ophthalmoplegia with pupillary involvement as an initial clinical presentation of herpes zoster ophthalmicus. J Am Osteopath Assoc.

[5] Harpaz R, Ortega-Sanchez IR, Seward JF; Advisory Committee on Immunization Practices (ACIP) (2008). Prevention of Herpes Zoster: Recommendations of the Advisory Committee on Immunization Practices (ACIP). MMWR Recomm Rep.

[6] Nicholas AS, Nicholas EA (2008). Atlas of osteopathic techniques.

[7] Marcdante KJ, Kliegman RM (2015). Nelson Essentials of Pediatrics, 7th ed. ..

[8] Massengill JS, Kittredge JL (2014). Practical considerations in the pharmacological treatment of postherpetic neuralgia for the primary care provider. J Pain Res.

[9] Pierce K, Wanat K, Egwuatu S (2018). Patterns and treatment of post-herpetic neuralgia: a case study. JAOCD.

[10] Goldman L, Schafer AI (2015). Goldman-Cecil Medicine, 25th ed. https://evolve.elsevier.com/cs/product/9781455750177?role=student.

[11] Shelley WB (1978). Surgical treatment for recurrent herpes simplex. Lancet.

[12] Hallaq IY, Harris JD (1969). The syndrome of postherpetic neuralgia: complication and an approach to therapy. J Am Osteopath Assoc.

[13] Coffey Coffey, D. D. (1995). AAO case study: trigeminal neuralgia. AAO J.

[14] Richardson BS, Way BV, Speece AJ 3rd (2009). Osteopathic manipulative treatment in the management of notalgia paresthetica. J Am Osteopath Assoc.

[15] Lewis DD, Summers GK (2017). Osteopathic manipulative treatment for the management of adjacent segment pathology. J Am Osteopath Assoc.

[16] Origo D, Tarantino AG (2019). Osteopathic manipulative treatment in pudendal neuralgia: A case report. J Bodyw Mov Ther.

